# Sequence-based bacterial small RNAs prediction using ensemble learning strategies

**DOI:** 10.1186/s12859-018-2535-1

**Published:** 2018-12-21

**Authors:** Guifeng Tang, Jingwen Shi, Wenjian Wu, Xiang Yue, Wen Zhang

**Affiliations:** 1 School of Computer Science, Wuhan University, Wuhan, 430072 China; 2 School of Mathematics and Statistics, Wuhan University, Wuhan, 430072 China; 3 Electronic Information School, Wuhan University, Wuhan, 430072 China; 4 Department of Computer Science and Engineering, The Ohio State University, Columbus, OH 43210 USA; 5 College of Informatics, Huazhong Agricultural University, Wuhan, 430070 China

**Keywords:** Small RNA prediction, Sequence-derived feature, Ensemble learning, Neural network

## Abstract

**Background:**

Bacterial small non-coding RNAs (sRNAs) have emerged as important elements in diverse physiological processes, including growth, development, cell proliferation, differentiation, metabolic reactions and carbon metabolism, and attract great attention. Accurate prediction of sRNAs is important and challenging, and helps to explore functions and mechanism of sRNAs.

**Results:**

In this paper, we utilize a variety of sRNA sequence-derived features to develop ensemble learning methods for the sRNA prediction. First, we compile a balanced dataset and four imbalanced datasets. Then, we investigate various sRNA sequence-derived features, such as spectrum profile, mismatch profile, reverse compliment k-mer and pseudo nucleotide composition. Finally, we consider two ensemble learning strategies to integrate all features for building ensemble learning models for the sRNA prediction. One is the weighted average ensemble method (WAEM), which uses the linear weighted sum of outputs from the individual feature-based predictors to predict sRNAs. The other is the neural network ensemble method (NNEM), which trains a deep neural network by combining diverse features. In the computational experiments, we evaluate our methods on these five datasets by using 5-fold cross validation. WAEM and NNEM can produce better results than existing state-of-the-art sRNA prediction methods.

**Conclusions:**

WAEM and NNEM have great potential for the sRNA prediction, and are helpful for understanding the biological mechanism of bacteria.

## Background

Non-coding RNAs (ncRNAs) are a class of RNA molecules that do not encode proteins. In general, non-coding RNA molecules are classified into three major types: ribosomal RNA (rRNA), messenger RNA (mRNA) and transfer RNA (tRNA). rRNA is the RNA component of the ribosome; mRNA is a messenger that delivers genetic information from DNA to the ribosome; tRNA is an adaptor molecule that has the capability of linking mRNA and the amino acid sequence of proteins. As a new kind of non-coding RNAs, small non-coding RNAs (sRNAs) have gained wide attention since the discovery of the first sRNA in bacteria.

The sRNAs have been detected in almost all kingdoms of life and they are high abundant during normal growth of cell [1]. The sRNAs are usually 50–500 nucleotides (nt) in length [2]. The majority of sRNAs regulate their target genes by base pairing and function as diffusible molecules [3]. Therefore, sRNAs can play important roles in controlling cellular processes in bacteria, such as cell proliferation, metabolic reactions and carbon metabolism [4]. Since sRNAs in bacteria have different functions, predicting sRNAs provides significance for understanding biological mechanisms. Wet lab methods identify sRNAs by using deep sequencing [5]. However, these methods are tremendously expensive, laborious and time-consuming. There exist a large number of unexplored sRNAs, which makes it impossible to identify sRNAs effectively through biochemical experiments.

In recent years, many computational methods have been proposed for the sRNA prediction. These methods are roughly classified as three types: comparative genomics methods, free energy methods and machine learning methods. Comparative genomics methods identify sRNAs by comparing sequence or structural homology to known sRNAs from different bacteria. Axmann [6] identified cyanobacteria non-coding RNAs by comparative genomics. Pichon [7] proposed a program named “Intergenic Sequence Inspector” (ISI) to identify sRNAs. Klein [8] developed a screening technique to predict sRNAs. Free energy methods utilize the free energy change when sRNA sequences transform into ordinary structure to distinguish sRNAs from pseudo sRNAs. Uzilov [9] predicted the second structure of sRNA by minimizing the folding free energy change. Machine learning methods transform the sRNA prediction as the binary classification problem. The binary classification methods take the real sRNAs as positive instances, and construct pseudo sRNAs as negative instances, and then formulate the work as the binary classification. Yachie [10] developed a gapped Markov model to predict non-coding and antisense RNA genes in *E. coli*. Tjaden [11] integrated primary sequence data, transcript expression data and conserved RNA structure information to predict sRNAs in bacteria via Markov models. Saetrom [12] used the sequence information to build sRNA classifiers by combining genetic programming and boosting algorithms. Arnedo [13] incorporated different existing sRNA prediction methods. Carter [14] utilized the composition information of sRNA sequences to train support vector machine (SVM) models and neural network (NN) models. Barman [15] used tri-nucleotide composition of sequences to construct SVM-based models. Generally, machine learning-based methods for the sRNA prediction have greater efficiency and better performances than comparative genomics methods and free energy methods. Besides, there are a number of successfully applications of machine learning techniques in bioinformatics [16–30].

Motivated by previous machine learning-based methods, we believe that there is still room to improve the sRNA prediction performances. One important point is how to make the best of various sRNA sequence-derived features, because sRNA sequences bring important information for the sRNA prediction. To the best of our knowledge, sequence-derived features have been used to successfully solve a large number of bioinformatics problems [31–38].

In this paper, we develop computational methods for the sRNA prediction by utilizing sRNA sequence-derived features, as the extension of our previous work [39]. Compared with existing methods, we consider diverse sRNA sequence-derived features to build prediction models. First of all, we compile one balanced dataset and four imbalanced datasets from the experimentally validated sRNAs of Salmonella Typhimurium LT2 (SLT2). Second, we investigate a variety of sRNA sequence-derived features, such as spectrum profile, mismatch profile, reverse compliment k-mer and pseudo nucleotide composition. Finally, two ensemble learning strategies are used to integrate diverse features. One is the weighted average ensemble method (WAEM), which uses the linear weighted sum of outputs from the individual sRNA feature-based predictors to predict sRNAs, and the genetic algorithm is adopted to optimize the parameters in the ensemble system. The other is the neural network ensemble method (NNEM), which trains neural networks in two steps by combining features from the same feature groups. In the 5-fold cross validation experiments, WAEM achieves AUC scores of 0.942, 0.952, 0.951, 0.957 and 0.957 on the balanced dataset and four imbalanced datasets, and NNEM produces AUC scores of 0.958, 0.962, 0.961, 0.962 and 0.961 on the five datasets. WAEM and NNEM outperform existing sRNA prediction methods. Moreover, our studies can reveal the importance of features in the sRNA prediction, and provide the guide to the wet experiments.

## Materials and methods

### Datasets

As far as we know, lots of experimentally validated sRNAs are publicly available. In this paper, we compiled our benchmark datasets from the sRNAs of Salmonella Typhimurium LT2 (SLT2) [40]. First, we downloaded the complete genome sequence of SLT2 in NCBI (http://www.ncbi.nlm.nih.gov/nuccore/16763390?report=fasta), and extracted 193 sRNA sequences according to the start and the end position information of the specific SLT2 sRNA provided in [41]. This data was used by Barman [15] and Arnedo [13]. Then, we removed 11 redundant sRNAs, and used the remaining 182 experimentally verified sRNAs as positive instances. Finally, we used EMBOSS shuffleseq program to randomly shuffle the complete genome sequence [42], and utilized the same position information to extract sequence fragments from the shuffled sequence. We used these sequence fragments as negative instances.

Actually, we can shuffle the complete genome sequence many times to obtain different negative instances datasets. To avoid the influence of data bias, we constructed one balanced dataset and four imbalanced datasets. The ratios of positive instances to negative instances are 1:1, 1:2, 1:3, 1:4 and 1:5, respectively. Table 1 summarizes five datasets used in this paper.

**Table 1:** Benchmark datasets of SLT2

Dataset	Ratio	#Positive instances	#Negative instances
Balanced	1:1	182	182
Imbalanced	1:2	182	364
	1:3	182	546
	1:4	182	728
	1:5	182	910

**Fig. 1:**
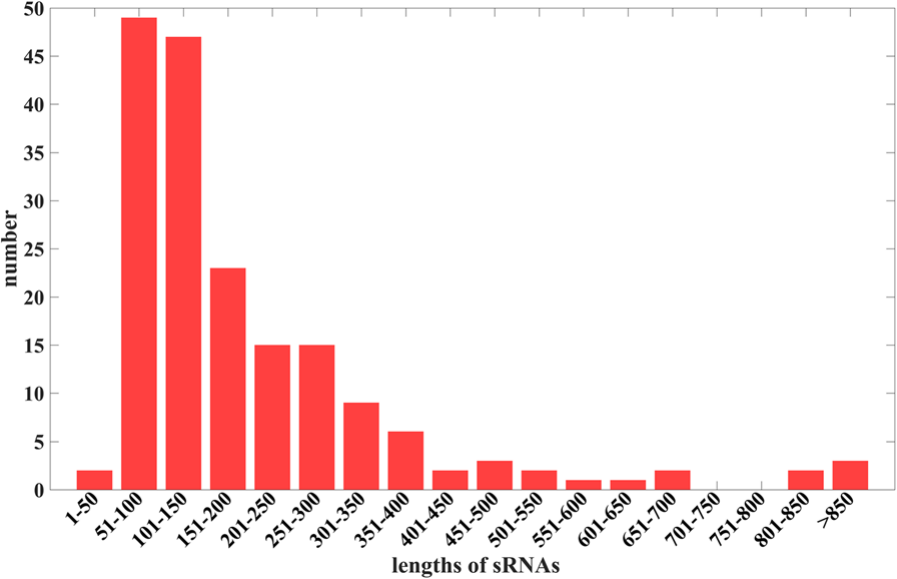
The length distribution of sRNAs in SLT2

Besides, we analyzed the length distribution of SLT2 sRNA sequences. Figure 1 demonstrates that lengths of sRNA sequences are significantly different. The majority of sRNA sequences have typical lengths that range from 45 nt to 250 nt, but some sRNA sequences may have more than 500 nucleotides. In our SLT2 sRNA dataset, the shortest sRNA sequence has 45 nucleotides.

### Sequence-derived features of sRNAs

The sRNA sequences have four types of nucleotides A, C, G and T, and their lengths are quite different. As far as we know, lots of RNA sequence-derived features have been proposed to characterize sRNAs, and several web servers and software [43–46] have been developed to extract features from sequences. In this work, we consider the following features for the sRNA prediction, and they are implemented by using the python package “repDNA” [43].

*k*-spectrum profile: *k*-spectrum profile is also known as *k*-mer profile. The spectrum profile describes repeated patterns of sequences. There are 4^*k*^ types of *k*-length contiguous subsequences, and the *k*-spectrum profile of a sRNA sequence is to count *k*-length contiguous subsequences [47]. Given a sequence *x*, the *k*-spectrum profile is defined as ${f}_k^{spe}(x)=\left({c}_1,{c}_2,\dots {c}_{4^k}\right)$, where *c*_*i*_ is the occurrence frequency of corresponding *k*-length contiguous subsequences. Spectrum profile has been widely adopted in biological applications [14, 15, 31, 32].

Mismatch profile: (*k, m*)-mismatch profile is similar to *k*-spectrum profile but allowing up to *m* (*m* < *k*) mismatches in the exact *k*-length contiguous subsequences [48, 49]. Given a sequence *x*, the (*k, m*)-mismatch profile is defined as ${f}_k^{mis}(x)=\left({\sum}_{j=0}^m{c}_{1j},{\sum}_{j=0}^m{c}_{2j},\dots, {\sum}_{j=0}^m{c}_{4^kj}\right)$, where *c*_*ij*_ denotes the occurrence frequency of the *i*th *k*-length contiguous subsequence with *j* mismatches.

Reverse compliment *k*-mer (*k*-RevcKmer): the feature is a kind of deformation of *k*-mer [43, 50], and it takes the reverse complement of RNA into consideration. Given a sequence *x*, the reverse complement *k*-length contiguous subsequences are removed after generating *k*-mer, then the occurrence frequencies of the remaining *k*-length subsequences are calculated to constitute a feature vector.

Pseudo nucleotide composition features: the feature contains occurrences of different di-nucleotides or tri-nucleotides as well as their physicochemical properties [43]. There are four types of pseudo nucleotide composition features: parallel correlation pseudo di-nucleotide composition (PCPseDNC), parallel correlation pseudo tri-nucleotide composition (PCPseTNC), series correlation pseudo di-nucleotide composition (SCPseDNC), and series correlation pseudo tri-nucleotide composition (SCPseTNC). The pseudo nucleotide composition features have a parameter *λ* representing the highest counted rank of the correlation along a sequence. More details about pseudo nucleotide composition features are described in [32, 43].

For the spectrum profile features, we considered the 1-spectrum profile, 2-spectrum profile, 3-spectrum profile, 4-spectrum profile and 5-spectrum profile. For the mismatch profile features, we considered the (3, *m*)-mismatch profile, (4, *m*)-mismatch profile and (5, *m*)-mismatch profile. For the reverse compliment *k*-mer features, we considered the 1-RevcKmer, 2-RevcKmer, 3-RevcKmer, 4-RevcKmer and 5-RevcKmer. For the pseudo nucleotide composition features, we considered PCPseDNC, PCPseTNC, SCPseDNC and SCPseTNC. All these features are demonstrated in Table 2.

**Table 2:** Sequence-derived features of sRNA

Feature group	Index	Feature	Dimension	Parameter
Spectrum profile	F1	1-spectrum profile	4	No parameter
	F2	2-spectrum profile	16	No parameter
	F3	3-spectrum profile	64	No parameter
	F4	4-spectrum profile	256	No parameter
	F5	5-spectrum profile	1024	No parameter
Mismatch profile	F6	(3, *m*)-mismatch profile	64	*m*: the max mismatches
	F7	(4*, m*)-mismatch profile	256	*m*: the max mismatches
	F8	(5*, m*)-mismatch profile	1024	*m*: the max mismatches
Reverse compliment k-mer	F9	1-RevcKmer	2	No parameter
	F10	2-RevcKmer	10	No parameter
	F11	3-RevcKmer	32	No parameter
	F12	4-RevcKmer	136	No parameter
	F13	5-RevcKmer	512	No parameter
Pseudo nucleotide composition	F14	PCPseDNC	16 + *λ*	*λ*: the highest counted rank
	F15	PCPseTNC	64 + *λ*	*λ*: the highest counted rank
	F16	SCPseDNC	16 + 6 × *λ*	*λ*: the highest counted rank
	F17	SCPseTNC	64 + 12 × *λ*	*λ*: the highest counted rank

### Ensemble learning strategies

Since there are various sequence-derived features, how to take full advantage of these features is critical for the sRNA prediction. In machine learning, ensemble learning is a useful technique which can integrate diverse features to produce better performances and generalization [51]. Studies have shown that ensemble learning can successfully solve a number of bioinformatics problems [52–60]. We develop two ensemble learning strategies for the sRNA prediction: the weighted average ensemble method (WAEM) and the neural network ensemble method (NNEM).

**Fig. 2:**
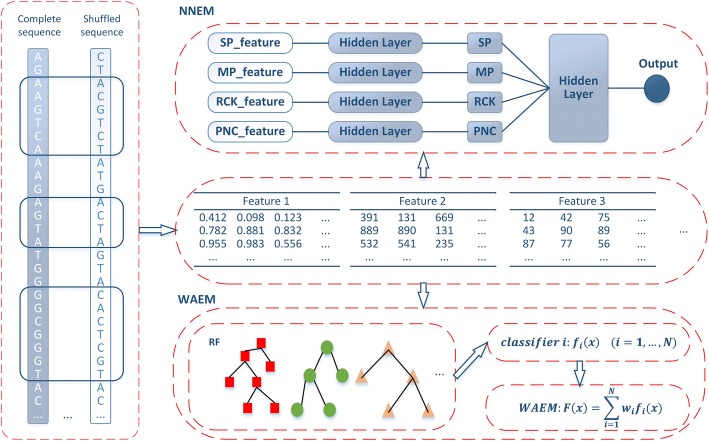
The workflow of WAEM and NNEM

Figure 2 shows the workflow of two ensemble learning methods. First, we obtain experimentally verified sRNAs of SLT2 and construct pseudo sRNAs to compile the benchmark datasets. Second, we extract various RNA sequence-derived features. Third, two ensemble learning strategies (WAEM and NNEM) are proposed to make the best of these features for the sRNA prediction.

### Weighted average ensemble method

As shown in Fig. 2, WAEM relies on the basic predictors and the weighted average ensemble rule. The basic predictors are the primary component in WAEM, and they can be constructed by using different features or different machine learning classifiers. Since we consider a variety of sequence-derived features, we adopt a suitable machine learning classifier to build basic predictors. Here, we compared two popular machine learning classifiers: random forest (RF) and support vector machine (SVM), and we adopted RF to construct individual sequence feature-based prediction models as basic predictors because of its high accuracy (results are provided in the section “Evaluation of features”).

We design a weighted average ensemble rule to combine the outputs of base predictors for the sRNA prediction. Given *N* features, we construct *N* base predictors *f*_*i*_(*i* = 1, 2, …*N*). For a new RNA sequence *x*, *f*_*i*_(*x*) represents the prediction probability of being predicted as a real sRNA by the base predictors *f*_*i*_. The final prediction probability of the sequence *x* is given by

1 $$ {\displaystyle \begin{array}{l}F(x)=\sum \limits_{i=1}^N{w}_i{f}_i(x)\\ {}\sum \limits_{i=1}^N{w}_i=1\kern0.5em ,\kern0.6em {w}_i\ge 0\end{array}} $$

Here, we consider the determination of weights as an optimization problem and solve it by the genetic algorithm (GA). GA is an intelligent search algorithm which simulates the biological evolution, and its capability for the optimization problems has been proved in many applications [31, 36].

In the GA optimization, our purpose is to search the max AUC score of WAEM on the training data. First, we randomly generate 100 weight vectors as the candidate solutions, and encode these candidates into chromosomes as the initial population. For a chromosome, we adopt the AUC score of NNEM as the fitness score. Then, we update the population by three operators, i.e., selection, crossover and mutation. The selection probability, crossover probability and mutation probability are dynamically adjusted according to the fitness scores of chromosomes [61]. After 200 generations of update, we determine optimal weights. Finally, the ensemble system makes predictions for the testing set.

### Neural network ensemble method

Artificial neural networks (ANNs) are popular prediction models inspired by the human brain. An ANN has a collection of connected nodes called artificial neurons, and the connection can transmit a signal from one neuron to another neuron. A multilayer perceptron (MLP) is a class of feedforward artificial neural networks. An MLP consists of an input layer, an output layer and one or more hidden layers. The information of input data is processed by activation functions in hidden layers and passed through to the units in each layer. MLP utilizes a supervised learning technique called backpropagation training algorithm for training. Here, we design a multilayer perceptron model (NNEM) to integrate diverse sRNA sequence-derived features.

Figure 2 presents the neural network architecture of NNEM. We merge features from the same feature groups to construct four types of feature vectors: SP features, MP features, RCK features and PNC features, and they are from feature group spectrum profile, mismatch profile, reverse compliment *k*-mer and pseudo nucleotide composition respectively. Then, we implement two steps to construct a NNEM model. In the first step, we build four MLP models by utilizing the four types of merged feature vectors, and our purpose is to integrate features from the same feature groups. The outputs of four MLP models are the node named “SP”, “MP”, “RCK” and “PNC” respectively. In the second step, we use outputs of four MLP models as inputs to build a MLP model, which can produce final predictions.

We utilize backpropagation training algorithm to train MLP models in NNEM. For NNEM, the parameters of MLP models are extremely important and they can determine the final performances. In the first step, the cross entropy and L2 regularization term are used as loss function. We use L2 regularization term because the lengths of the feature vectors are greater than the sizes of datasets. We use the python package “scikit-learn” to implement four MLP models. We adopt one hidden layer whose activation function is the “relu” function. The size of the four hidden layers are all set to 700 and the L2 regularization term parameter is set to 0.3. In the second step, we use cross entropy as loss function. We implement the MLP model by using Tensorflow. Similarly, we use one hidden layer, which has 10 nodes, and adopt the “relu” activation function.

### Evaluation metrics

In this paper, we estimate performances of prediction models by 5-fold cross validation (5-CV). In the 5-CV, the whole dataset is randomly divided into 5 equal-sized subsets and each subset is constructed by means of stratified sampling from the dataset. Then four subsets are combined as the training set, and the remaining subset is used as the testing set in each fold of 5-CV. We construct prediction models on the training set and then make predictions for the testing set. The process of training and testing is performed until each subset has been used for testing, and averaged performances over five folds are adopted as overall performances of models.

Here, we adopt several common performance metrics of binary classification problem to evaluate performances of the proposed method. According to the real labels and the predicted labels, instances can be divided into four classes: true positive (TP), false positive (FP), true negative (TN) and false negative (FN). Therefore, four metrics: sensitivity (SN), specificity (SP), accuracy (ACC) and AUC score are defined as follows.

$$ SN=\frac{TP}{TP+ FN} $$

$$ SP=\frac{TN}{TN+ FP} $$

$$ ACC=\frac{TP+ TN}{TP+ FP+ TN+ FN} $$

The AUC score is the area under receiver operating characteristic curve (ROC) which is plotted by using the false positive rate (1-specificity) against the true positive rate (sensitivity) for different cutoff thresholds. Clearly, the larger the AUC score is, the better the predictor performs. We adopt the AUC score as the primary metric because it assesses the performance regardless of any threshold.

## Results and discussion

### Parameters of features

As shown in Table 2, among all seventeen sRNA sequence-derived features we consider, the features in mismatch profile feature group and pseudo nucleotide composition feature group have parameters. For better prediction in the following study, it is requisite to discuss how to set parameters in the computational experiments.

For mismatch profile feature group, the parameter *k* means the length of contiguous subsequences and *m* represents the max mismatches. Commonly, *m* is set to less than one-third of *k*. In this paper, *m* is set to 3, 4 and 5, therefore, we consider (3, 1)-mismatch profile, (4, 1)-mismatch profile and (5, 1)-mismatch profile.

**Fig. 3:**
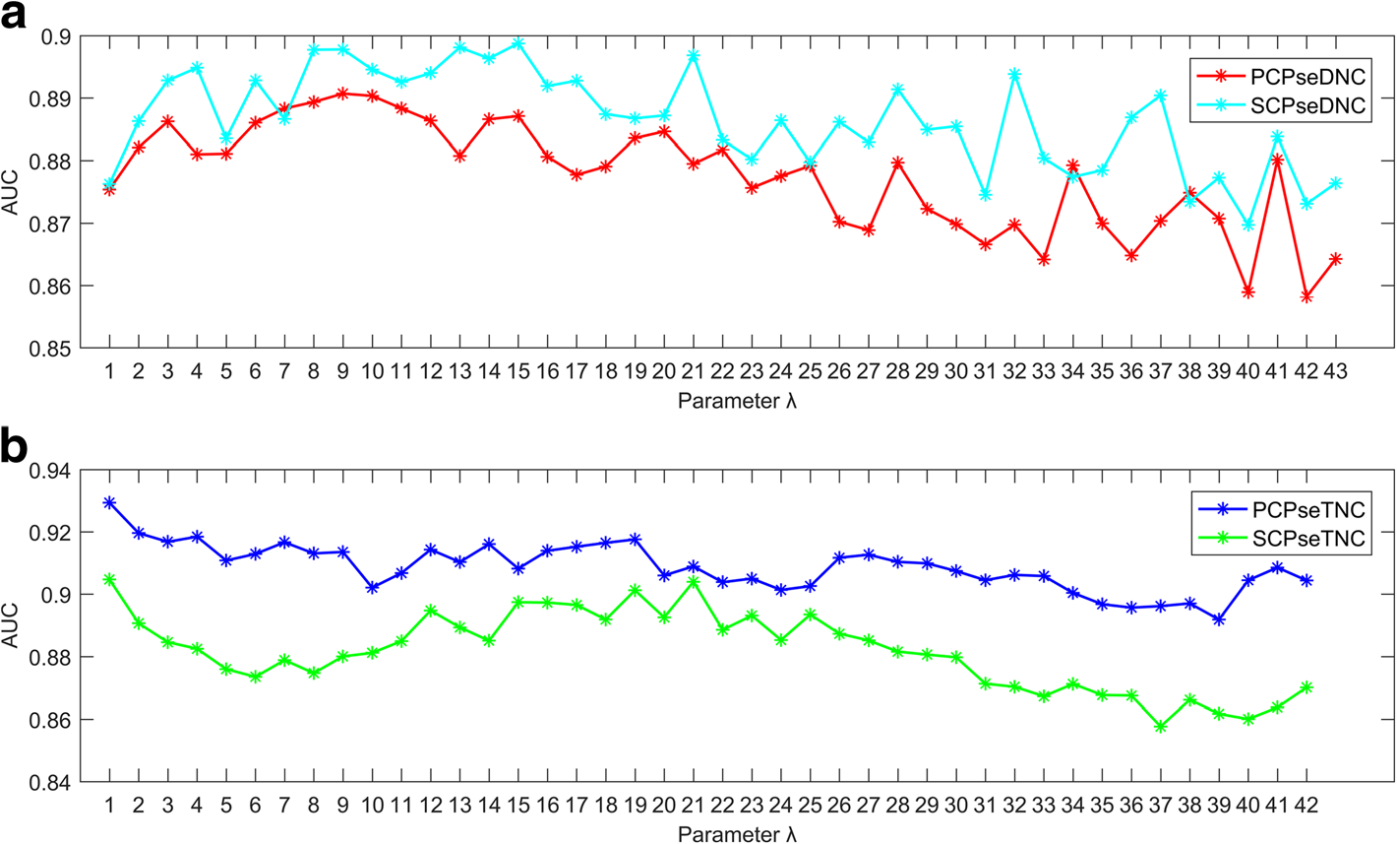
**a** AUC scores of the PCPseDNC and SCPseDNC-based models with the variation of the parameter λ on the balanced dataset; **b** AUC scores of the PCPseTNC and SCPseTNC-based models with the variation of the parameter λ on the balanced dataset

For pseudo nucleotide composition feature group, the parameter *λ* is an integer which means the highest counted rank of the correlation along a sequence. In PCPseDNC and SCPseDNC, *λ* ranges from 1 to *L* − 2. In PCPseTNC and SCPseTNC, *λ* ranges from 1 to *L* − 3. *L* is the length of the shortest sRNA sequence, and is 45 according to the section “Datasets”. To select the best parameter *λ* on the four features, we evaluated the four features with different parameters on the balanced dataset by using 5-fold cross validation, and random forest was used to construct prediction models. As shown in Fig. 3 (a) and Fig. 3 (b), when *λ* was set to 9, 15, 1 and 1, PCPseDNC, SCPseDNC, PCPseTNC and SCPseTNC could achieve the greatest AUC scores. Therefore, we used these values for pseudo nucleotide composition in the following study.

### Evaluation of features

For the comprehensive study, we compared the capabilities of the sequence-derived features in Table 2 for the sRNAs prediction. We constructed individual feature-based models and implemented 20 runs of 5-fold cross validation on the five benchmark datasets in the section “Datasets”.

First of all, to test different machine learning classifiers, we constructed models on the balanced dataset by using random forest (RF) and support vector machine (SVM). Here, we respectively implemented RF and SVM by using python “scikit-learn” package. For RF, we set the number of trees in the forest to 200 and used the default value for other parameters. For SVM, we tested different kernels and adopted RBF kernel due to its better performance. As shown in Table 3, RF outperforms SVM on twelve features in terms of AUC scores. Hence, we adopted RF as the classification engine to build prediction models in the following study.

**Table 3:** Performances of individual feature-based models constructed by RF and SVM on the balanced dataset

Index	Feature	AUC		ACC		SN		SP	
		RF	SVM	RF	SVM	RF	SVM	RF	SVM
F1	1-spectrum profile	0.682	0.657	0.560	0.512	0.912	0.985	0.209	0.039
F2	2-spectrum profile	0.829	0.821	0.756	0.749	0.792	0.788	0.720	0.711
F3	3-spectrum profile	0.909	0.874	0.834	0.800	0.863	0.835	0.805	0.765
F4	4-spectrum profile	0.923	0.909	0.860	0.840	0.873	0.866	0.846	0.814
F5	5-spectrum profile	0.912	0.896	0.842	0.822	0.847	0.874	0.838	0.770
F6	(3, *m*)-mismatch profile	0.769	0.795	0.679	0.717	0.807	0.812	0.552	0.622
F7	(4*, m*)-mismatch profile	0.880	0.885	0.797	0.816	0.814	0.843	0.780	0.789
F8	(5*, m*)-mismatch profile	0.913	0.907	0.835	0.832	0.848	0.882	0.822	0.782
F9	1-RevcKmer	0.632	0.655	0.516	0.542	0.972	0.935	0.060	0.150
F10	2-RevcKmer	0.842	0.804	0.765	0.726	0.828	0.817	0.702	0.636
F11	3-RevcKmer	0.924	0.868	0.855	0.791	0.848	0.831	0.863	0.750
F12	4-RevcKmer	0.938	0.894	0.880	0.818	0.880	0.869	0.880	0.768
F13	5-RevcKmer	0.937	0.906	0.874	0.829	0.859	0.856	0.889	0.802
F14	PCPseDNC	0.895	0.905	0.827	0.828	0.850	0.868	0.803	0.787
F15	PCPseTNC	0.931	0.922	0.862	0.857	0.856	0.848	0.868	0.865
F16	SCPseDNC	0.902	0.888	0.825	0.811	0.841	0.810	0.809	0.811
F17	SCPseTNC	0.905	0.910	0.825	0.840	0.854	0.841	0.795	0.839

Furthermore, to test the influences of ratios of positive instances vs. negative instances on performances of prediction models, we constructed models by using RF on the five benchmark datasets. As shown in Table 4, different prediction models may produce similar performances on different benchmark datasets, which indicates that these sequence-derived features are robust to the data ratio. In general, most features can produce high-accuracy results on the balanced dataset and four imbalanced datasets. Among the seventeen features, 4-spectrum profile (F4), 4-RevcKmer (F12) and PCPseTNC (F15) features have better performances than other features for the sRNA prediction. Since different features can bring different information and no features have the extremely poor performances. Therefore, we adopted all features to build the ensemble learning systems.

**Table 4:** Performances of individual feature-based models constructed by RF on the benchmark datasets

Index	AUC					ACC				
	Balanced	Imbalanced				Balanced	Imbalanced			
	1:1	1:2	1:3	1:4	1:5	1:1	1:2	1:3	1:4	1:5
F1	0.682	0.718	0.730	0.729	0.738	0.560	0.691	0.754	0.804	0.840
F2	0.829	0.847	0.862	0.865	0.868	0.756	0.789	0.836	0.863	0.877
F3	0.909	0.917	0.921	0.928	0.930	0.834	0.856	0.887	0.905	0.915
F4	0.923	0.933	0.930	0.934	0.933	0.860	0.884	0.906	0.921	0.930
F5	0.912	0.894	0.872	0.869	0.863	0.842	0.864	0.882	0.896	0.910
F6	0.769	0.808	0.822	0.832	0.840	0.679	0.766	0.809	0.843	0.866
F7	0.880	0.902	0.910	0.917	0.922	0.797	0.842	0.870	0.894	0.909
F8	0.913	0.924	0.929	0.938	0.939	0.835	0.871	0.901	0.916	0.927
F9	0.632	0.657	0.667	0.679	0.691	0.516	0.619	0.707	0.755	0.791
F10	0.842	0.847	0.865	0.875	0.875	0.765	0.796	0.836	0.867	0.882
F11	0.924	0.926	0.933	0.941	0.944	0.855	0.879	0.901	0.920	0.930
F12	0.938	0.949	0.948	0.954	0.954	0.880	0.902	0.918	0.931	0.942
F13	0.937	0.932	0.923	0.924	0.920	0.874	0.897	0.910	0.925	0.936
F14	0.895	0.883	0.886	0.888	0.884	0.827	0.805	0.835	0.864	0.876
F15	0.931	0.922	0.922	0.924	0.921	0.862	0.855	0.876	0.895	0.902
F16	0.902	0.894	0.890	0.890	0.887	0.825	0.833	0.859	0.882	0.897
F17	0.905	0.898	0.901	0.903	0.899	0.825	0.822	0.854	0.877	0.897

### Performances of ensemble methods

In this section, we evaluated the performances of the weighted average ensemble method (WAEM) and the neural network ensemble method (NNEM) by implementing 20 runs of 5-fold cross validation on the five benchmark datasets.

As shown in Table 5, WAEM achieves AUC score of 0.942 on the balanced dataset and outperforms the best-performed individual feature-based model, which is based on 4-RevcKmer feature (F12) and produces the AUC score of 0.938. Similarly, WAEM performs accurate prediction on the datasets with imbalance ratios 1:2, 1:3, 1:4 and 1:5, and achieves AUC scores of 0.952, 0.951, 0.957 and 0.957 respectively. WAEM also performs better than individual feature-based predictors on the four imbalanced datasets. The results demonstrate that WAEM has not only high-accuracy performances but also good robustness.

**Table 5:** Performances of WAEM and NNEM on the balanced and imbalanced datasets

Dataset	Ratio	Method	AUC	ACC	SN	SP
Balanced	1:1	WAEM	0.942	0.887	0.888	0.868
		NNEM	0.958	0.901	0.903	0.899
Imbalanced	1:2	WAEM	0.952	0.901	0.853	0.925
		NNEM	0.962	0.909	0.872	0.927
	1:3	WAEM	0.951	0.915	0.818	0.948
		NNEM	0.961	0.920	0.819	0.954
	1:4	WAEM	0.957	0.929	0.817	0.956
		NNEM	0.962	0.931	0.810	0.961
	1:5	WAEM	0.957	0.934	0.808	0.959
		NNEM	0.961	0.940	0.782	0.972

**Fig. 4:**
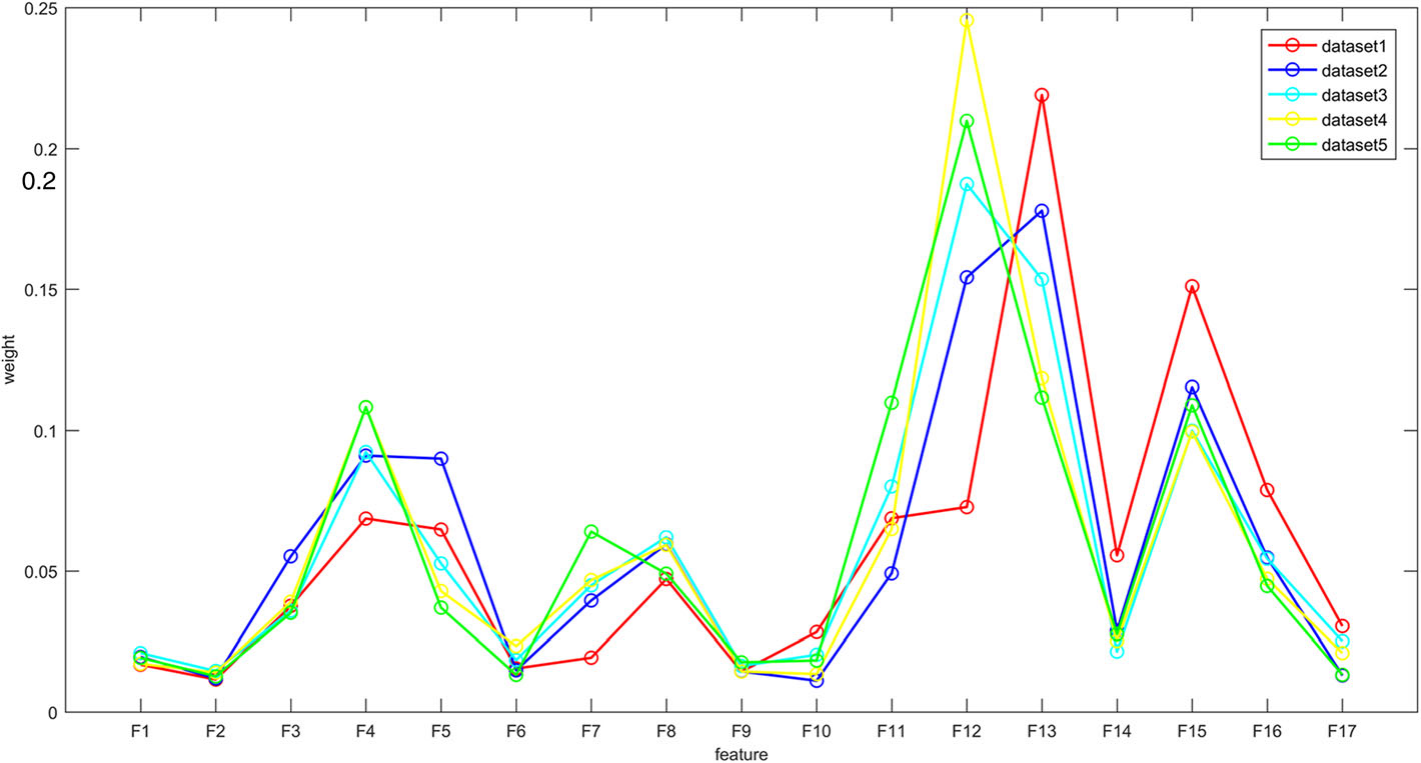
Optimal weights for the WAEM models on the benchmark datasets. dataset1 means balanced dataset 1:1, dataset2 means imbalanced dataset 1:2, dataset3 means imbalanced dataset 1:3, dataset4 means imbalanced dataset 1:4, dataset5 means imbalanced dataset 1:5

We analyzed the optimal weights for individual feature-based predictors (base predictors) in different datasets. Weights in WAEM on the balanced dataset and four imbalanced datasets are visualized in Fig. 4. As we can see, weights for individual feature-based predictors are different, and no weight is equal to zero. The contributions of individual feature-based predictors to WAEM are reflected by the corresponding weights. Therefore, we can conclude that every individual feature-based predictor is useful for improving the performance of predicting SRNAs. From Table 4, we know that the base predictors based on the 4-RevcKmer (F12), 5-RevcKmer (F13) and PCPseTNC (F15) features have the best performances among all predictors, and thus 4-RevcKmer, 5-RevcKmer and PCPseTNC have greater weights than other features, indicating they make more contributions to WAEM models. This is consistent with our expectations. At the same time, WAEM can automatically determine the weights for base predictors, and has the good interpretability.

As we can see from Table 5, NNEM produces the AUC scores of 0.958, 0.962, 0.961, 0.962 and 0.961 on the five benchmark datasets. The performance of NNEM is better than that of the individual feature-based predictors, indicating that the network-based ensemble strategy can effectively combine diverse information to improve performances. NNEM also produces better performances than WAEM. Further, we tested the statistical difference between WAEM and NNEM. Table 6 displays the *P*-values, which are obtained through paired t-test of AUCs of WAEM and NNEM on five benchmark datasets. The result demonstrates that NNEM is significantly better than WAEM on all five benchmark datasets (*P*-value< 0.05). The possible reason is that the linear ensemble learning strategy in WAEM cannot deal with complicated data and the neural ensemble learning strategy is more suitable for our task.

**Table 6:** P-values of paired t-test on the AUCs of WAEM and NNEM on benchmark datasets

Dataset	Balanced	Imbalanced			
	1:1	1:2	1:3	1:4	1:5
P-values	1.67E-09	3.07E-06	7.26E-09	1.12E-05	5.70E-03

### Comparison with existing sRNA prediction methods

To the best of our knowledge, several state-of-the-art machine learning-based computational methods have been proposed to predict sRNAs. Here, we adopted the latest methods Carter’ s method [14] and Barman’s method [15] for comparison. Carter built SVM models to identify sRNAs by utilizing mono-nucleotide composition and di-nucleotide composition. Actually, mono-nucleotide composition and di-nucleotide composition are same as the 1-spectrum profile and 2-spectrum profile which are used in our models. Barman also adopted SVM to predict sRNAs by using tri-nucleotide composition, which are 3-spectrum profile in this paper.

We respectively built different prediction models based on the balanced dataset and four imbalanced datasets. All models were evaluated by 5-CV. As shown in Table 7, the AUC scores of NNEM, WAEM, Barman’s method and Carter’s method are 0.958, 0.942, 0.938 and 0.566 on the balanced dataset respectively. Compared with Barman’s method and Carter’s method, NNEM’s average AUC scores are 2.1 and 69.3% higher and WAEM’s average AUC scores are 0.4 and 66.4% higher. WAEM and NNEM also yield much better ACC scores than Barman’s method and Carter’s method. Moreover, WAEM and NNEM produce greater AUC scores and ACC scores on four imbalanced datasets than Barman’s method and Carter’s method. All results demonstrate that WAEM and NNEM are more powerful than Barman’s method and Carter’s method for the sRNA prediction. There are several reasons why WAEM and NNEM have excellent prediction performances. First, we consider seventeen sRNA sequence-derived features in our models rather than one or two features in the other models, and this can guarantee the information diversity. Second, we utilize a more efficient classifier to build basic predictors. Finally, the ensemble learning strategies provide an efficient way to integrate a variety of features for the better sRNA predicting performances.

**Table 7:** Performances of different methods on benchmark datasets

Dataset	Ratio	Method	AUC	ACC	SN	SP
Balanced	1:1	Carter’s method	0.566	0.511	0.264	0.758
		Barman’s method	0.938	0.882	0.846	0.918
		WAEM	0.942	0.887	0.888	0.868
		NNEM	0.958	0.901	0.903	0.899
Imbalanced	1:2	Carter’s method	0.602	0.678	0.033	1.000
		Barman’s method	0.937	0.884	0.851	0.916
		WAEM	0.952	0.901	0.853	0.925
		NNEM	0.962	0.909	0.872	0.927
	1:3	Carter’s method	0.619	0.757	0.030	1.000
		Barman’s method	0.944	0873	0.818	0.927
		WAEM	0.951	0.915	0.818	0.948
		NNEM	0.961	0.920	0.819	0.954
	1:4	Carter’s method	0.627	0.805	0.025	1.000
		Barman’s method	0.944	0.874	0.818	0.929
		WAEM	0.957	0.929	0.817	0.956
		NNEM	0.962	0.931	0.810	0.961
	1:5	Carter’s method	0.636	0.835	0.011	1.000
		Barman’s method	0.943	0.875	0.884	0.865
		WAEM	0.957	0.934	0.808	0.959
		NNEM	0.961	0.940	0.782	0.972

## Conclusions

Bacterial small non-coding RNAs are regarded as important regulators and play essential roles in controlling diverse physiological processes. Predicting sRNAs is an important and challenging topic, which provides clues for understanding the biological mechanism of bacteria. This paper is aimed to design the computational method for the sRNA prediction. We consider various sRNA sequence-derived features. Then we propose two ensemble learning methods (WAEM and NNEM) to integrate diverse features for the sRNA prediction. Experimental results based on the benchmark SLT2 datasets show that WAEM and NNEM can produce high-accuracy performances when evaluated by 5-fold cross validation. By fair comparison on same datasets, WAEM and NNEM outperform state-of-the-art methods. In conclusion, the methods we proposed are promising tools for predicting sRNAs in bacteria.

## Abbreviations

5-CV: 5-fold cross validation
AUC: area under ROC curve
GA: genetic algorithm
MLP: multilayer perceptron
NNEM: neural network ensemble method
PCPseDNC: parallel correlation pseudo di-nucleotide composition
PCPseTNC: parallel correlation pseudo tri-nucleotide composition
RF: random forest
SCPseDNC: series correlation pseudo di-nucleotide composition
SCPseTNC: series correlation pseudo tri-nucleotide composition
SLT2: Salmonella Typhimurium LT2
SVM: support vector machine
WAEM: weighted average ensemble method
